# α-Glucosidase inhibitor miglitol attenuates glucose fluctuation, heart rate variability and sympathetic activity in patients with type 2 diabetes and acute coronary syndrome: a multicenter randomized controlled (MACS) study

**DOI:** 10.1186/s12933-017-0571-1

**Published:** 2017-07-06

**Authors:** Michio Shimabukuro, Atsushi Tanaka, Masataka Sata, Kazuoki Dai, Yoshisato Shibata, Yohei Inoue, Hiroki Ikenaga, Shinji Kishimoto, Kozue Ogasawara, Akira Takashima, Toshiyuki Niki, Osamu Arasaki, Koichi Oshiro, Yutaka Mori, Masaharu Ishihara, Koichi Node, on behalf of the Collaborators on the Effect of Miglitol on Glucose Metabolism in Acute Coronary Syndrome (MACS) Study

**Affiliations:** 1 Department of Diabetes, Endocrinology, and Metabolism, Fukushima Medical University, Fukushima, Japan; 2 Department of Cardio-Diabetes Medicine, Institute of Biomedical Sciences, Tokushima University Graduate School, Tokushima, Japan; 3 Department of Cardiovascular Medicine, Saga University, Saga, Japan; 4 Department of Cardiovascular Medicine, Institute of Biomedical Sciences, Tokushima University Graduate School, Tokushima, Japan; 5 Department of Cardiology, Hiroshima City Hospital, Hiroshima, Japan; 6 Miyazaki Medical Association Hospital, Cardiovascular Center, Miyazaki, Japan; 7 Department of Cardiology, Tomishiro Central Hospital, Okinawa, Japan; 8 Department of Cardiology, Ohama Dai-ichi Hospital, Okinawa, Japan; 9 Division of Diabetes and Endocrinology, Department of Internal Medicine, Jikei University School of Medicine, Tokyo, Japan; 10 Division of Coronary Heart Disease, Department of Internal Medicine, Hyogo College of Medicine, Nishinomiya, Japan

**Keywords:** Acute coronary syndrome, Glucose fluctuation, Heart rate variability, Hypoglycemia, Miglitol, Sympathetic nervous system activity, Type 2 diabetes

## Abstract

**Background:**

Little is known about clinical associations between glucose fluctuations including hypoglycemia, heart rate variability (HRV), and the activity of the sympathetic nervous system (SNS) in patients with acute phase of acute coronary syndrome (ACS). This pilot study aimed to evaluate the short-term effects of glucose fluctuations on HRV and SNS activity in type 2 diabetes mellitus (T2DM) patients with recent ACS. We also examined the effect of suppressing glucose fluctuations with miglitol on these variables.

**Methods:**

This prospective, randomized, open-label, blinded-endpoint, multicenter, parallel-group comparative study included 39 T2DM patients with recent ACS, who were randomly assigned to either a miglitol group (n = 19) or a control group (n = 20). After initial 24-h Holter electrocardiogram (ECG) (Day 1), miglitol was commenced and another 24-h Holter ECG (Day 2) was recorded. In addition, continuous glucose monitoring (CGM) was performed throughout the Holter ECG.

**Results:**

Although frequent episodes of subclinical hypoglycemia (≤4.44 mmo/L) during CGM were observed on Day 1 in the both groups (35% of patients in the control group and 31% in the miglitol group), glucose fluctuations were decreased and the minimum glucose level was increased with substantial reduction in the episodes of subclinical hypoglycemia to 7.7% in the miglitol group on Day 2. Holter ECG showed that the mean and maximum heart rate and mean LF/HF were increased on Day 2 in the control group, and these increases were attenuated by miglitol. When divided 24-h time periods into day-time (0700–1800 h), night-time (1800–0000 h), and bed-time (0000–0700 h), we found increased SNS activity during day-time, increased maximum heart rate during night-time, and glucose fluctuations during bed-time, which were attenuated by miglitol treatment.

**Conclusions:**

In T2DM patients with recent ACS, glucose fluctuations with subclinical hypoglycemia were associated with alterations of HRV and SNS activity, which were mitigated by miglitol, suggesting that these pathological relationships may be a residual therapeutic target in such patients.

*Trial registration* Unique Trial Number, UMIN000005874 (https://upload.umin.ac.jp/cgi-open-bin/ctr_e/ctr_view.cgi?recptno=R000006929)

**Electronic supplementary material:**

The online version of this article (doi:10.1186/s12933-017-0571-1) contains supplementary material, which is available to authorized users.

## Introduction

It is well established that hyperglycemia on admission to hospital is associated with increased mortality in patients with acute myocardial infarction (AMI) [1, 2]. Recent studies have shown that hypoglycemia is also associated with increased short- and long-term mortality in AMI patients [3–5]. Lee et al. [6] compared the prognostic significance of hypoglycemia and hyperglycemia in 34,943 AMI patients with or without type 2 diabetes mellitus (T2DM) from Korean registries. They found that admission hypoglycemia in patients with poorly controlled T2DM was associated with a markedly decreased 30-day survival rate. Nevertheless, HbA1c level was not associated with the risk of 30-day mortality.

Although the mechanism by which hypoglycemia leads to increased mortality is not clear, an abnormal QT prolongation during severe hypoglycemia may be associated with increased 30-day mortality [7]. In experimentally-induced hypoglycemia, abnormal QT prolongation, sinus bradycardia, and ventricular arrhythmias occurred that were potentially fatal [8, 9]. A clinical study has described bradycardia during severe hypoglycemia in both diabetic and non-diabetic patients, which may reflect imbalance of the sympathetic nervous system (SNS) [7, 8]. However, associations between hypoglycemia, heart rate variability (HRV), and the activity of the SNS have not been studied in patients with AMI/acute coronary syndrome (ACS).

Alpha-glucosidase inhibitors (αGIs) are a class of oral anti-diabetic drugs that act by inhibiting the hydrolysis of complex carbohydrates into glucose. Although they lower the degree of postprandial hyperglycemia, they also reduce the degree of hypoglycemia, probably by diminishing postprandial hyperinsulinemia [10]. Therefore, we hypothesized that αGI drugs stabilize glucose fluctuations, and thereby decrease the number of hypoglycemic episodes, in consequence lessening variation(s) in heart rate (HR) and/or imbalance of the SNS.

In this multicenter randomized controlled trial, we evaluated the effects of glucose fluctuations on HRV and autonomic nervous system activity in T2DM patients with ACS. We also investigated the effects of the αGI miglitol on glucose fluctuations and the other variables.

## Methods

### Study design

The effect of miglitol on glucose metabolism in acute coronary syndrome (MACS) study is a multicenter prospective, randomized, open-label, blinded endpoint (PROBE) pilot study. The protocol was approved by the local Ethics Committees and carried out in accordance with the principles of the Declaration of Helsinki. All subjects gave written informed consent. This trial has been registered in the University Hospital Medical Information Network Clinical Trials Registry (UMIN000005874).

### Patients recruitment

A total of 39 T2DM patients with recent ACS were recruited at five sites in Japan after obtaining consent, and were randomly allocated to two groups with (miglitol group) or without (control group) miglitol per os, 50 mg three times daily. Inclusion criteria were as follows: (1) ACS for <7 days from onset; (2) fasting plasma glucose ≥6.99 mmol/L or plasm glucose ≥11.10 mmol/L either postprandially or in two-hourly measurements during a 75 g oral glucose tolerance test (OGTT); (3) HbA1c <9.4% (79.2 mmol/mol); (4) men or women aged ≥20 and <80 years; and (5) written informed consent. ACS was diagnosed as: (1) chest pain within 24-h of admission that lasted for >30 min. and was not relieved by sublingual nitroglycerin; (2) ST-segment elevation and/or an abnormal Q-wave on electrocardiogram (ECG); and (3) elevated serum creatine kinase. Exclusion criteria were as follows: (1) type 1 diabetes mellitus or diabetes treated with insulin or α-GI medication; (2) severe liver disease, including carrier status of hepatitis B or C; (3) severe renal disease, including serum creatinine ≥176.8 µmol/L; (4) a history of gastrointestinal surgery; (5) a history of drug allergy; (7) pregnancy or possible pregnancy.

All patients received coronary angiography on admission and then underwent percutaneous coronary intervention (PCI) using coronary stents. Patients were routinely treated with heparin, isosorbide dinitrate, clopidogrel, aspirin, a statin, and an angiotensin-converting enzyme inhibitor or angiotensin II receptor blocker. All the patients were managed in accordance with Japanese guidelines for rehabilitation in patients with cardiovascular disease [11]. After hemodynamic and general conditions had been stabilized within 7 days of the onset of ACS, all patients gave informed consent to participate in the trial. A 2-h OGTT was performed in patients who had no previous diagnosis of diabetes and/or HbA1c <6.5% (47.5 mmol/mol).

### Randomization and study intervention

**Fig. 1:**
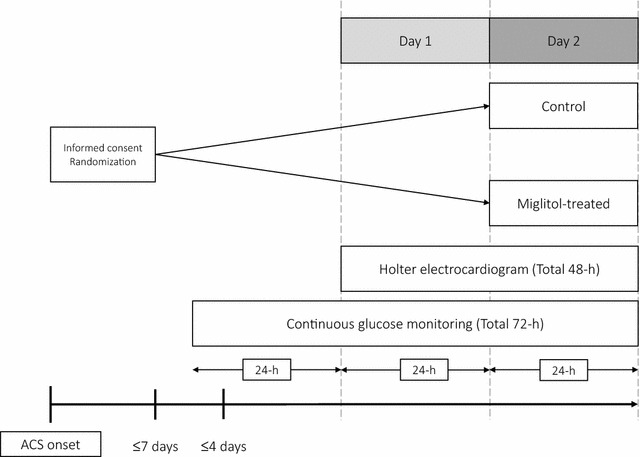
Study outline. *ACS* acute coronary syndrome

Consent was obtained 2–7 days after PCI due to ACS at each local site, and patients were registered at the administration office of the MACS study (Department of Cardiovascular Medicine, Saga University). Thereafter, patients were randomly assigned to the miglitol group or the control group (1:1) using a non-biased table of random numbers. The CGM test was initiated within the next 4 days. During 72-h continuous glucose monitoring (CGM) (Fig. 1), background medication was continued and no new treatment was started, in principle. If patients took oral antidiabetic agents and/or β-blockers, their doses were not changed during the study. In the miglitol group, treatment was initiated after an initial 48-h of CGM examination.

### Study outcomes

The study was a post hoc exploratory analysis of the MACS pilot study. We evaluated short-term effects of glucose fluctuations on HRV and SNS activity in T2DM patients after recent onset of ACS. In addition, we studied the effects of miglitol on the variables.

### Monitoring of continuous blood glucose and heart rate variability

In all participants, simultaneous monitoring of glucose and Holter ECG was performed. Within 4-day of informed consent, patients underwent CGM using the CGMS (Medtronic iPro2) [12], and Holter ECG monitoring was introduced 24-h after the start of CGM. CGM and Holter ECG were recorded during the next 48-h in parallel by synchronizing the timing of the devices. Both were recorded without pharmacological treatment during the first 24-h (Day 1), and then continued with or without miglitol *per os* 50 mg three times daily during the second 24-h (Day 2) (Fig. 1). Blood glucose levels were calibrated by simultaneous finger-stick tests at least four times daily. A standard hospital diet, which contained ~25 kcal/kg/day × ideal body weight (15% protein, 23–25% fat and 60–62% glucose), was provided at around 0800 h (8 a.m.), 1200 h (noon), and 1800 h (6 p.m.).

Intra-day glycemic variability was expressed as the mean amplitude of glycemic excursions (MAGE), which was calculated according to Service et al. [13] by measuring the arithmetic mean of differences between the consecutive peaks and nadirs, if the differences exceeded the standard deviation around the mean glucose level. Measurements of the peak-to-nadir or nadir-to-peak directions were determined by the first qualifying excursion. Hypoglycemia was defined as glucose ≤4.44 mmol/L in the CGM system [14]. Hermanns et al. [14] reported that if the point of care (POC) measurement of hypoglycemic blood glucose showed a plasma glucose of ≤3.89 mmol/L, the CGM system had a glucose measurement ≤4.44 mmo/L in interstitial fluid. The CGM data from 1 patient in the control group and four miglitol-treated patients were excluded from the analysis due to recording failure.

Assessment of HRV was carried out using the parameters recommended by the working group of the European Society of Cardiology as follows: standard deviation of normal RR intervals (SDNN); the root mean square of the differences in successive pairs of RR intervals (RMSSD); the ratio between the power of low frequency (LF) and high frequency (HF) bands (LF/HF) [15]. To evaluate the measured values according to the time of day, the daily records were allocated in three time slots from 0700 to 1800 h (day-time), 1800–0000 h (night-time) and 0000–0700 h (bed-time).

### Blood sampling and analysis

Peripheral venous blood and urine samples were collected from all subjects before the start of miglitol or control treatment. Plasma samples were collected into ethylenediamine tetra-acetic acid anticoagulant. Plasma and urinary samples were stored at −80 °C until required for analysis. Plasma glucose concentrations were determined by the glucose oxidase method. HbA1c was determined by high-performance liquid chromatography, and 1,5-anhydro-d-glucitol (1,5-AG) was assayed by an enzymatic method. Serum total cholesterol and triglyceride were determined by routine enzymatic methods. High-density lipoprotein cholesterol was measured by an enzymatic method after heparin and calcium precipitation.

### Statistical analysis

Statistical analysis was performed using JMP software (version 12.2.0, SAS Institute Inc., Cary, NC, USA). The variables were expressed as mean ± SD or %. Data between two groups were compared using the un-paired Student’s *t* test. Variables between Day 1 and Day 2 were compared using the paired Student’s *t* test for parametric variables or Wilcoxon signed-rank test for non-parametric variables. Data for categorical variables were analyzed with Fisher’s exact test. *P* < 0.05 was considered to be statistically significant.

## Results

### General characteristics

**Fig. 2:**
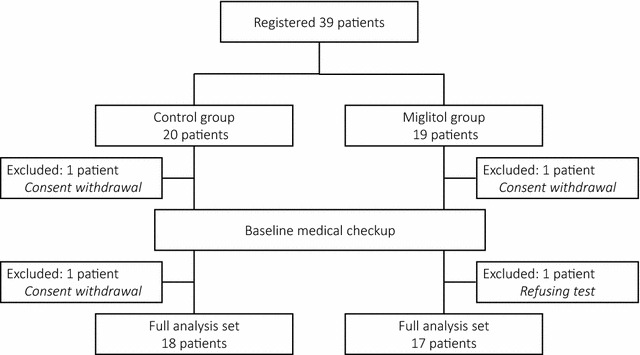
Participants’ flow

A total of 39 participants were randomly assigned to either the miglitol group (*n* = 19) or the control group (*n* = 20). Prior to the assessment of baseline characteristics, two participants were excluded due to withdrawal of consent. Subsequently, another two participants dropped out as a consequence of withdrawal of consent or refusing to be tested prior to the initiation of CGM test. Seventeen in the miglitol group and 18 in the control group were included in the full analysis (Fig. 2).

Baseline characteristics did not differ between two groups: the control group included 15 men and 4 women with a mean age of 63 ± 13 years, and the miglitol group included 16 men and 2 women with a mean age of 62 ± 12 years. At baseline, body weight, body mass index, blood pressure, HR, and left ventricular ejection fraction were all comparable between the two groups (Table 1). Blood levels of glucose, HbA1c, 1,5-AG, lipids, and creatinine were also comparable between groups. During CGM and Holter ECG, oral antidiabetic agents (including metformin, gliclazide, glimepiride or pioglitazone) and ß-blockers (including bisoprolol and carvedilol) had been taken equally in the control group (15 and 55%, respectively) and the miglitol group (16 and 63%, respectively), and these drugs were not changed during the study. All participants tolerated the treatment with no reported major adverse events.

**Table 1:** General characteristics of patients in the two groups

Parameters	Control (*n* = 19)	Miglitol-treated (*n* = 18)	*P* value
Men (%)	15 (79)	16 (89)	0.659
Age (years)	63 (13)	62 (12)	0.855
Body weight (kg)	69.9 (13.8)	70.9 (15.7)	0.855
Body mass index (kg/m^2^)	26.2 (4.7)	25.3 (4.3)	0.536
Systolic blood pressure (mmHg)	119 (20)	116 (19)	0.665
Diastolic blood pressure (mmHg)	68 (10)	67 (10)	0.866
Heart rate (beats/min)	75 (14)	76 (10)	0.794
Left ventricular ejection fraction (%)	53 (13)	54 (10)	0.760
Fasting plasma glucose (mmol/L)	7.94 (2.94)	7.83 (1.78)	0.870
HbA1c (%)	6.5 (1.1)	6.8 (0.9)	0.357
HbA1c (mmol/mol)	47.5 (11.5)	50.8 (13.7)	0.357
1,5-anhydro-d-glucitol (µg/mL)	16.0 (10.5)	12.3 (8.1)	0.240
Total cholesterol (mmol/L)	4.55 (0.93)	4.91 (1.19)	0.329
LDL cholesterol (mmol/L)	2.74 (0.75)	2.92 (0.93)	0.534
HDL cholesterol (mmol/L)	1.19 (0.34)	1.32 (0.36)	0.300
Triglyceride (mmol/L)	1.35 (0.61)	1.47 (0.98)	0.673
Non-HDL cholesterol (mmol/L)	3.36 (0.85)	3.59 (1.19)	0.528
Creatinine (µmol/L)	73.4 (17.7)	75.1 (18.6)	0.751

Values are mean (SD) or *n* (%)

*LDL* low-density lipoprotein, *HDL* high-density lipoprotein

### Continuous monitoring of blood glucose

**Fig. 3:**
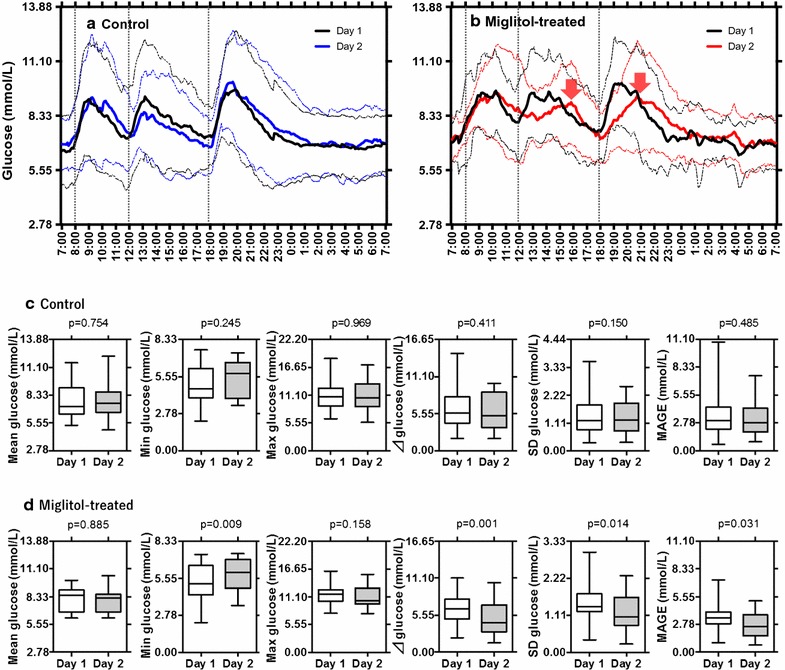
Continuous glucose monitoring. **a**, **b** Mean variations in 24-h blood glucose measured by a continuous glucose monitoring system in control and miglitol groups. *Lines* represent mean (*solid*) ± SD (*dotted*) of blood glucose levels on Day 1 (*black*) and Day 2 (*blue* or *red*) in **a** control (*n* = 17) and **b** miglitol (*n* = 13) groups. To see daily variations of glucose levels, we divided time periods into 0700–1800 h (day-time), 1800–0000 h (night-time), and 0000–0700 h (bed-time). Patients took a standard regimen of breakfast, lunch, and supper. The peaks after lunch and supper on Day 2 in the miglitol group (*red arrow*, Fig. 3b) are indicated by *red arrows*. **c**, **d** Levels of mean glucose, minimum (min) glucose, maximum (max) glucose, ∆glucose, SD glucose, and MAGE during 24-h in the control and miglitol groups.* Boxes* represent mean ± SD, and whiskers represent min to max during 24-h on Day 1 and Day 2 in **c** control (*n* = 17) and **d** miglitol (*n* = 13) groups

**Fig. 4:**
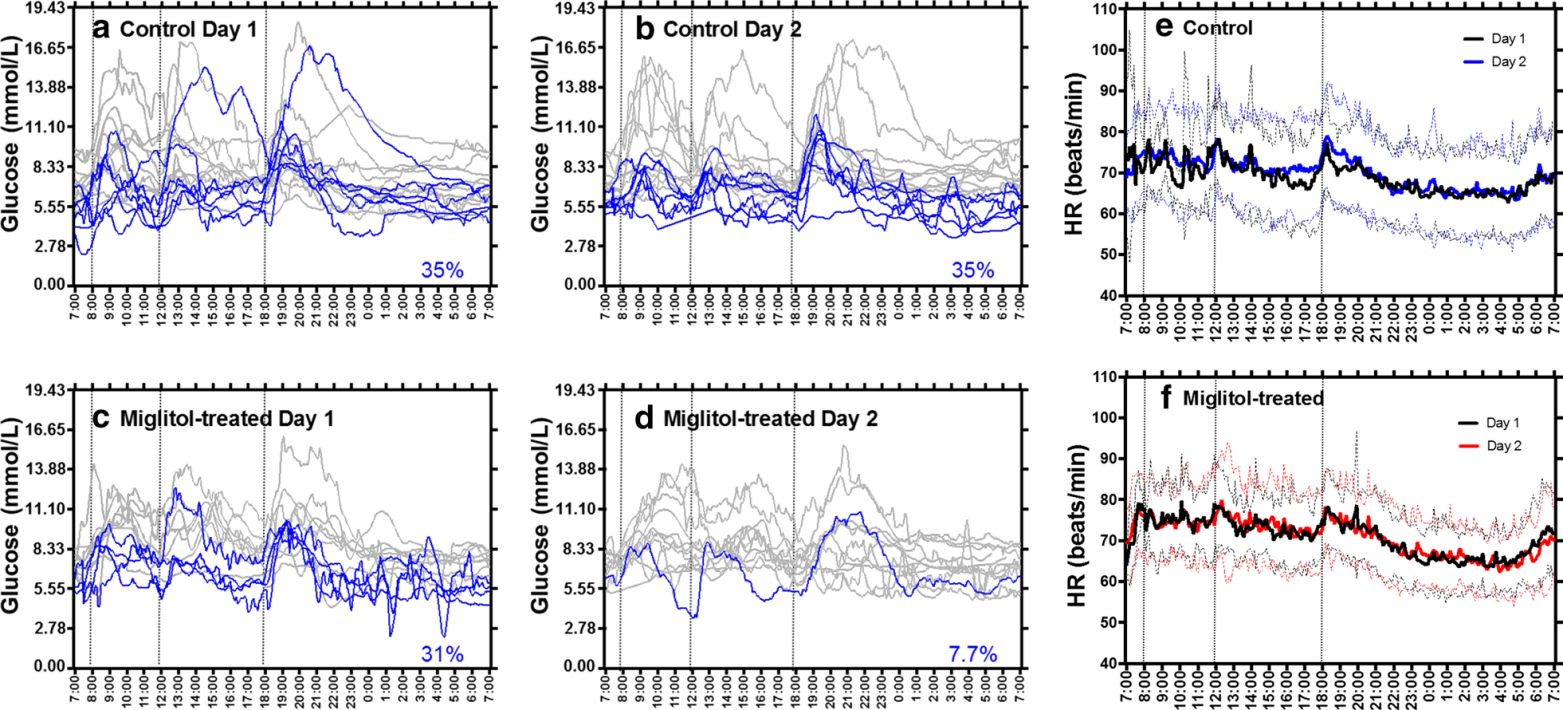
Impact of miglitol on glucose fluctuation, heart rate variability, and sympathetic nervous activity. **a**–**d** Plots of 24-h blood glucose measured by a continuous monitoring system in control and miglitol-treated patients. *Lines* indicate blood glucose levels on Day 1 and Day 2 in (**a**, **c**) control (*n* = 17) and (B, D) miglitol (*n* = 13) groups. To see daily variations of glucose levels, we divided time periods into 0700–1800 h (day-time), 1800–0000 h (night-time), and 0000–0700 h (bed-time). Patients took a standard regimen of breakfast, lunch and supper. The percentage values indicate the proportions of patients with episodes of glucose ≤4.44 mmol/L throughout the day, indicated by *blue lines* on the figures. **e**, **f** Mean variations in 24-h heart rate (HR) in the control and miglitol groups. *Lines* represent mean (solid) ± SD (*dotted*) of mean heart rate measured by Holter electrocardiography on Day 1 (*black*) and Day 2 (*blue* or *red*) in **e** control (*n* = 18) and **f** miglitol (*n* = 17) groups. To see daily variations of heart rate, we divided time periods into 0700–1800 h (day-time), 1800–0000 h (night-time), and 0000–0700 h (bed-time)

Mean variations in 24-h blood glucose levels and blood glucose indices measured by CGM in the control and miglitol groups are shown in Fig. 3. The postprandial peaks of blood glucose after breakfast, lunch, and supper were comparable between Day 1 and Day 2 in the control group (Fig. 3a), while they slowed after lunch and supper on Day 2 as compared to Day 1 in the miglitol group (Fig. 3b). Results for mean glucose, minimum (min) glucose, maximum (max) glucose, ∆glucose, SD glucose, and MAGE during 24 h were comparable between Day 1 and Day 2 in the control group (Fig. 3c), while results for mean and max glucose were comparable, min glucose was higher, and ∆glucose, SD glucose and MAGE were lower on Day 2 in the miglitol group (Fig. 3d). To see daily variations of blood glucose levels, we divided 24-h time periods into 0700–1800 h (day-time), 1800–0000 h (night-time), and 0000–0700 h (bed-time) (Tables 2, 3). In these three time periods, the control group showed an increase in the levels of ∆glucose during bed-time on Day 2. The miglitol group showed significant decreases in the max, ∆, and SD glucose during night-time, and an increase in the min glucose during bed-time. During the 24-h CGM, hypoglycemic episodes (defined by blood glucose ≤4.44 mmol/L) occurred in 35% (6/17) of patients (Day 1) and 35% (6/17) of patients (Day 2) in the control group (p = 0.554) (Fig. 4a, b), while the number was reduced from 31% (4/13) (Day 1) to 7.7% (1/13) (Day 2) in the miglitol group (p = 0.089) (Fig. 4c, d). The control group had hypoglycemic episodes as follows: 35% (6/17) of patients (Day 1) and 12% (2/17) of patients (Day 2) during day-time; 12% (2/17) (Day 1) and 18% (3/17) (Day 2) during night-time; and 6% (1/17) (Day 1) and 29% (5/17) (Day2) during bed-time. The miglitol group showed hypoglycemic episodes as follows: 31% (4/13) (Day 1) and 7.7% (1/13) (Day 2) during day-time; 7.7% (1/13) (Day 1) and 7.7% (1/13) (Day 2) during night-time; 23% (3/13) (Day 1) and 0% (0/13) (Day 2) during bed-time.

**Table 2:** Glucose and heart rate variability during 24-h and each time-phase in the control group

Parameters	Control (*n* = 17 in CGM, 18 in Holter ECG)											
	0700–0700 h (24-h)			0700–1800 h (day-time)			1800–0000 h (night-time)			0000–0700 h (bed-time)		
	Day 1	Day 2	*P* value	Day 1	Day 2	*P* value	Day 1	Day 2	*P* value	Day 1	Day 2	*P* value
Mean glucose (mmol/L)	7.66 (1.79)	7.72 (1.85)	0.758	7.91 (2.06)	7.77 (1.93)	0.640	8.25 (2.24)	8.50 (2.19)	0.296	6.77 (1.51)	6.96 (1.67)	0.508
Min glucose (mmol/L)	5.04 (1.47)	5.44 (1.35)	0.245	5.38 (1.57)	5.81 (1.10)	0.242	6.22 (1.53)	6.35 (1.44)	0.567	5.96 (1.48)	5.95 (1.72)	0.963
Max glucose (mmol/L)	11.24 (3.18)	11.26 (3.26)	0.969	10.62 (3.08)	10.28 (3.17)	0.333	10.52 (3.18)	10.85 (3.12)	0.411	7.68 (1.90)	8.17 (2.13)	0.189
∆ glucose (mmol/L)	6.21 (3.12)	5.82 (2.87)	0.411	5.24 (2.84)	4.47 (2.61)	0.056	4.30 (2.79)	4.50 (2.83)	0.453	1.72 (1.04)	2.22 (1.43)	0.045
SD glucose (mmol/L)	1.45 (0.87)	1.28 (0.69)	0.149	1.37 (0.85)	1.12 (0.67)	0.061	1.29 (0.86)	1.29 (0.87)	0.939	0.46 (0.30)	0.56 (0.38)	0.200
CV glucose (%)	18.3 (8.4)	16.4 (7.5)	0.175	16.3 (9.2)	13.8 (5.8)	0.190	14.0 (9.3)	15.0 (10.6)	0.493	8.3 (5.4)	10.0 (6.9)	0.200
Mean HR (beats/min)	68 (9)	70 (10)	0.002	69 (9)	70 (10)	0.157	66 (9)	66 (10)	0.168	66 (9)	66 (10)	0.400
Min HR (beats/min)	58 (8)	58 (8)	0.166	61 (10)	61 (8)	0.837	59 (8)	59 (9)	0.949	59 (8)	59 (9)	0.864
Max HR (beats/min)	89 (14)	93 (16)	0.012	85 (12)	89 (15)	0.152	82 (12)	87 (16)	0.015	82 (12)	87 (16)	0.074
SDNN (ms)	99 (35)	115 (42)	0.004	93 (38)	98 (30)	0.384	87 (33)	104 (41)	0.022	76 (30)	93 (40)	0.031
RMSSD (ms)	71 (29)	79 (34)	0.015	80 (40)	81 (31)	0.272	68 (27)	76 (36)	0.130	66 (24)	80 (39)	0.034
Mean LF/HF	1.11 (0.76)	1.24 (0.67)	0.044	0.97 (0.75)	1.21 (0.61)	0.012	1.15 (0.74)	1.22 (0.70)	0.312	1.28 (0.90)	1.28 (0.88)	0.968
Min LF/HF	0.04 (0.07)	0.08 (0.08)	0.093	0.09 (0.16)	0.11 (0.10)	0.608	0.13 (0.10)	0.17 (0.16)	0.118	0.12 (0.12)	0.12 (0.10)	0.989
Max LF/HF	6.77 (4.54)	8.17 (5.91)	0.188	4.18 (2.85)	6.97 (5.34)	0.013	4.84 (3.10)	5.78 (4.77)	0.245	6.03 (4.59)	5.75 (3.84)	0.618

Values are mean (SD)

*CGM* continuous glucose monitoring, *ECG* electrocardiogram, *SD* standard deviation, *CV* coefficient of variation, *HR* heart rate, *SDNN* standard deviation of normal RR intervals, *RMSSD* root mean square of the differences in successive pairs of RR intervals, *LF* low frequency, *HF* high frequency

**Table 3:** Glucose and heart rate variability during 24-h and each time-phase in the miglitol group

Parameters	Miglitol-treated (n = 13 in CGM, 17 in Holter ECG)											
	0700–0700 h (24-h)			0700–1800 h (day-time)			1800–0000 h (night-time)			0000–0700 h (bed-time)		
	Day 1	Day 2	*P* value	Day 1	Day 2	*P* value	Day 1	Day 2	*P* value	Day 1	Day 2	*P* value
Mean glucose (mmol/L)	8.66 (2.40)	8.69 (2.62)	0.867	8.55 (3.64)	9.25 (3.29)	0.872	9.24 (3.11)	8.84 (2.40)	0.330	7.29 (1.54)	7.67 (2.10)	0.220
Min glucose (mmol/L)	5.31 (1.93)	6.02 (1.37)	0.025	6.53 (2.22)	6.44 (1.25)	0.832	6.68 (2.32)	6.61 (1.47)	0.868	5.70 (2.02)	6.71 (1.90)	0.024
Max glucose (mmol/L)	12.39 (3.52)	11.87 (3.80)	0.157	11.84 (3.60)	11.30 (3.86)	0.121	11.78 (3.78)	10.73 (3.74)	0.019	8.74 (2.00)	9.08 (2.89)	0.405
∆ glucose (mmol/L)	7.08 (3.07)	5.85 (3.58)	0.004	4.92 (2.41)	4.51 (3.48)	0.450	4.73 (2.50)	3.82 (2.89)	0.019	3.04 (1.69)	2.37 (1.96)	0.192
SD glucose (mmol/L)	1.68 (0.90)	1.42 (1.00)	0.025	1.37 (0.63)	1.26 (0.95)	0.435	1.53 (0.70)	1.15 (0.80)	0.005	0.76 (0.45)	0.54 (0.45)	0.068
CV glucose (%)	18.9 (6.4)	15.6 (7.3)	0.007	16.8 (11.2)	12.8 (6.2)	0.154	17.0 (12.9)	12.5 (13.1)	0.055	13.7 (8.1)	9.8 (8.1)	0.068
Mean HR (beats/min)	71 (6)	71 (8)	0.643	69 (20)	74 (8)	0.893	71 (7)	71 (8)	0.968	66 (7)	66 (7)	0.423
Min HR (beats/min)	59 (7)	59 (7)	0.888	63 (6)	63 (8)	0.521	62 (7)	63 (8)	0.336	60 (7)	59 (7)	0.482
Max HR (beats/min)	96 (12)	95 (13)	0.916	93 (8)	95 (13)	0.461	90 (14)	87 (10)	0.246	86 (9)	83 (12)	0.139
SDNN (ms)	96 (37)	108 (43)	0.028	89 (35)	94 (32)	0.277	87 (36)	96 (38)	0.077	77 (33)	86 (36)	0.120
RMSSD (ms)	70 (30)	80 (35)	0.012	79 (42)	81 (32)	0.285	67 (27)	75 (37)	0.098	65 (24)	73 (36)	0.111
Mean LF/HF	1.09 (0.42)	1.14 (0.45)	0.421	1.05 (0.66)	1.10 (0.56)	0.589	1.04 (0.55)	1.11 (0.57)	0.174	1.08 (0.49)	1.05 (0.57)	0.653
Min LF/HF	0.06 (0.09)	0.08 (0.13)	0.154	0.12 (0.19)	0.12 (0.20)	0.972	0.12 (0.12)	0.14 (0.16)	0.174	0.13 (0.16)	0.15 (0.17)	0.575
Max LF/HF	6.39 (3.09)	5.78 (2.58)	0.469	3.88 (3.03)	4.46 (2.36)	0.446	3.77 (2.50)	4.05 (2.30)	0.641	4.99 (2.50)	4.51 (2.86)	0.535

Values are mean (SD). Abbreviations see Table 2

### Heart rate variability

Twenty-four-hour variations in HR measured by Holter ECG are shown in Fig. 4e (control) and Fig. 4f (miglitol-treated). HR, SDNN, RMSSD, and LF/HF during 24-h, day-time, night-time, and bed-time are shown in Tables 2 and 3. In the control group, min HR during 24-h was comparable between Day 1 and Day 2, but mean HR, max HR, SDNN, and RMSSD were increased on Day 2. During day-time, mean HR, min HR, max HR, SDNN, and RMSSD were not different between Day 1 and Day 2, but max HR during night-time, SDNN during night- and bed-time, and RMSSD during bed-time were increased, respectively (Table 2). On the other hand, in the miglitol group, mean HR, min HR, and max HR throughout the day were comparable on Day 1 and Day 2. In addition, SDNN and RMSSD were significantly increased during 24-h (Table 3).

In the control group, mean LF/HF during 24-h was increased, and mean and max LF/HF during day-time were significantly increased on Day 2. While, in the miglitol group, LF/HF indices during 24-h and even each time phase were all comparable between Day 1 and Day 2. Both ∆glucose and SD glucose during 24-h were correlated with mean LF/HF, but not with SDNN and RMSSD, in the miglitol group (Additional files 1, 2).

## Discussion

The new findings of this pilot study are: (1) T2DM patients with recent ACS manifested a substantial number of episodes of hypoglycemia (blood glucose ≤4.44 mmo/L) during the first 24-h CGM (Day 1). However, in the miglitol group, MAGE was significantly decreased on Day 2, and the min glucose level was increased and the episodes of hypoglycemia tended to be fewer after miglitol on Day 2; (2) mean and max HR during 24-h were increased on Day 2 in the control group, but the increases on Day 2 were mitigated in the miglitol group. Simultaneously, the increase in mean LF/HF during 24-h and day-time in the control group were also suppressed in the miglitol group. This is the first study to evaluate the associations between glucose fluctuations including hypoglycemia, HR and SNS activity in T2DM patients who had recently developed ACS. The study found that there is a substantial number of subclinical episodes of hypoglycemia in ACS and that they can be attenuated by diminishing the glucose fluctuations with a αGI, accompanied by concurrent reductions of HRV and autonomic nervous system activity.

There have been no published studies of 24-h continuous glucose monitoring in ACS patients. However, previous reports have suggested that there may be a substantial number of subclinical episodes of hypoglycemia in ACS patients. Lee et al. [6] reported that AMI patients had admission glucose levels <3.89 mmol/L in 1.20% (255/21,096) of diabetic patients and 0.73% (154/13,156) of non-diabetic patients. On Day 1 in the current study, diabetic patients with ACS had blood glucose levels ≤4.44 mmol/L in 35% (6/17) of participants in the control group and 31% (4/13) in the miglitol group. Although there are several differences in the protocols between our study and that of Lee et al. (admission vs post-admission, one-point vs continuous, and plasma glucose level vs interstitial blood glucose level estimated by CGMS), the high frequency of subclinical hypoglycemia in our study is not so questionable. Hermanns et al. [14] reported that if the POC measurement of hypoglycemic plasma showed a glucose value of ≤3.89 mmol/L, the CGM system gave a glucose level ≤4.44 mmol/L in interstitial fluid. Accordingly, we adopted ≤4.44 mmol/L in the CGM as a hypoglycemic alert. The value for the min glucose was increased and the frequency of episodes of blood glucose ≤4.44 mmol/L tended to be lower in the miglitol group on Day 2. It has been reported that αGI not only lowers the degree of postprandial hyperglycemia, but also decreases the degree of hypoglycemia, probably by diminishing postprandial hyperinsulinemia [10]. It is conceivable that αGI stabilizes glucose fluctuations, thereby decreasing the frequency of hypoglycemic episodes.

In the control group, the average of 24-h min HR was comparable between Day 1 and Day 2, but mean and max HR were increased on Day 2. Jabre et al. [16] reported that an increase in resting HR after the onset of ACS was a strong predictor of cardiovascular (CV) mortality. Although it has been suggested that elevated resting HR is an intrinsic risk factor for poor outcomes after MI rather than merely a marker of other CV risk factors, the underlying mechanisms by which resting HR increased are unknown [16]. Although we could not determine the mechanism, our study has shown that mean and max HR were increased on Day 2 in the control group. Since the standard protocol on cardiac rehabilitation in Japan allows bathing and walking freely in the hospital 6 days after PCI [11], a day-to-day increase in the in-hospital activity may influence their HR on Day 2. After being divided into three time periods, the max HR was increased only during night-time. The fact that max HR could be attenuated by miglitol suggests that HR may be partly affected by a postprandial or post-supper phase-dependent cause. Koivikko et al. [17] reported that marked and prolonged hypoglycemia increases the HR and decreases the high-frequency oscillations of HR. In our control group, the increases in the SNS activity during day-time and the max HR during night-time were accompanied by the increases in both glucose fluctuation (∆glucose ranged from 1.71 to 2.20 mmo/L) and hypoglycemic episodes (6–29%) during bed-time on Day 2. While, the miglitol treatment significantly increased min glucose and reduced glucose fluctuations and hypoglycemic episodes during bed-time, accompanied by attenuation of both increase of mean and max HR and SNS activation. These results suggest that impaired hemodynamic and neurohormonal parameters may be, in part, associated with the increases in both glucose fluctuation and hypoglycemic episodes, possibly forming a vicious cycle in T2DM patients with recent ACS. In addition, these changes may contribute to the intrinsic disturbance of hemodynamic and neurohormonal systems, partly resulting in a short-term poor prognosis after onset of ACS [1–6].

αGI mono- and combination therapy can delay glucose uptake in the intestine and lower the postprandial glucose spike with infrequent development of hypoglycemia in patients with T2DM or impaired glucose tolerance [10, 18, 19]. Kitano et al. [20] reported that short-term miglitol treatment in patients with postprandial hyperglycemia and recent ACS attenuated the postprandial glucose spike, accompanied by improvement of endothelial function. In addition, a previous large randomized controlled trial and a meta-analysis showed that αGI treatment could prevent the development of CV events [21, 22]. However, the precise mechanism underlying αGI-induced improvement of CV outcomes is yet to be determined. Moreover, little is known about miglitol-mediated effects on glucose fluctuations, HRV, and SNS activity in the acute phase of ACS. In the present study, miglitol exerted prompt beneficial effects on glucose fluctuations and reduced the number of hypoglycemic episodes. Furthermore, miglitol attenuated increases in the mean and max HR and the mean LF/FH throughout the day. Although the reason why glucose fluctuations parameters were not associated with time-domain HRV parameters remains to be explained, our results suggest that glucose fluctuations may be, at least partially, associated with the autonomic nerve activity. Given that oscillating glucose is associated with excess oxidative stress in vascular endothelial cells and consequent endothelial dysfunction [23], it is possible that suppression of oscillating glucose might contribute to CV protection after onset of ACS. Further studies would be required to evaluate possible association between glucose fluctuation and hemodynamics in the other classes of anti-hyperglycemic agents [24].

Our study has several limitations. First, although we detected extensive individual differences in HR and cardiac autonomic regulation during 24 h, it was a post hoc exploratory analysis of a small sample of a PROBE design study, but not a placebo-controlled study. Because unexpected bias might be introduced in the assessment of outcome, it may be difficult to reach strong clinical conclusion. In addition, we could not refer to the generalization of the current findings for other ethnicities because of no recruitment of the other ethnicities than Japanese, although previous studies demonstrated a common association between reduced HRV and impairment of glycemic metabolisms in Hispanic/Latino and Japanese adults [25, 26]. Although HRV parameters are influenced by physical and exercise intensity, those intensity was not compared between the groups. In addition, although guidelines for clinical use of HRV recommended that time-domain methods should be used when using long-term ECGs in clinical studies [27], but not frequency domain methods, time-domain parameters, such as SDNN and RMSSD, during 24-h were increased in both groups. Furthermore, the SDNN during night- and bed-time increased significantly only in the control group, although why such increases were less evident in the miglitol group remains to be clarified. In addition, several parameters on HRV and SNS activity increased during 24-h on Day 2 in the control group, while the time phases of their increases differed for HR, SDNN, and LH/FH, with the mean and max LF/HF increasing during day-time, and max HR increasing during night-time. Therefore, further trials equipped with more appropriate design and sample would be needed in the future. Second, the study was unable to determine if there was a cause-and-effect relationship. Although we obtained evidence that glucose fluctuations can be associated with changes in HR and HRV, we cannot determine whether glucose fluctuations, especially periods of hypoglycemia, can directly alter HR and HRV. We obtained no data on biomarkers of inflammation or oxidative stress during the study period. Third, because the aim of present study was to evaluate the effects of glucose fluctuations on HRV and SNS activity in T2DM patients with recent ACS, we did not recruit non-diabetic patients in this study. Accordingly, we could not elucidate enough the specific peculiarities of HRV in diabetes. Accumulated evidence suggest that disturbed HRV was associated with metabolic syndrome, insulin resistance and diabetes [28, 29], and it is also reported that glycemic variability was independently associated with the presence of cardiovascular autonomic neuropathy in patients with uncontrolled T2DM [30]. Therefore, further studies are also needed to elucidate the diabetes-specific peculiarities of HRV and the effective medical care. Fourth, the study protocol focused on only the short-term acute-phase after ACS. Hence, we cannot extrapolate the miglitol-mediated beneficial effect to the chronic-phase. However, our study provides novel observations during the acute-phase of ACS that may partly contribute to future improvement of CV outcomes. Fifth, hyperinsulinemia and insulin resistance may be related to glucose fluctuation and/or incidence of hypoglycemia especially in diabetic patients with obesity or visceral fat obesity, and miglitol can diminish it [31]. Although we did not measure beta cell function and insulin sensitivity in the current study, hyperinsulinemia/insulin resistance and/or leptin resistance may be related to glucose fluctuation and/or incidence of hypoglycemia. Sixth, the CGM system measured glucose levels in the interstitial fluid, not in plasma or in the capillary artery. Therefore, our data about daily glucose variations should be confirmed by serial measurements of plasma glucose levels.

In summary, this is the first study to evaluate the associations between glucose fluctuations, HRV, and SNS activity in T2DM patients with recent ACS. Subclinical hypoglycemia was more prevalent in ACS than expected. Even short-term treatment of miglitol could alter favorably HRV, SNS activity, and glucose fluctuation with avoiding hypoglycemic episodes. Disturbed hemodynamic and neurohormonal activities may be, in part, associated with increased glucose fluctuations and hypoglycemic episodes, suggesting a potential new strategy to improve outcomes in patients with recent ACS.

## Additional files

**Additional file 1.** Correlations of difference (∆Glucose) in 24-hour glucose levels with heart rate variability parameters.

**Additional file 2.** Correlations of standard deviation (SD Glucose) in 24-hour glucose levels with heart rate variability parameters.

## Abbreviations

ACS: acute coronary syndrome
αGIs: alpha-glucosidase inhibitors
AMI: acute myocardial infarction
CGM: continuous glucose monitoring
CV: cardiovascular
ECG: electrocardiogram
HF: high frequency
HR: heart rate
HRV: heart rate variability
LF: low frequency
MAGE: mean amplitude of glycemic excursions
OGTT: oral glucose tolerance test
PCI: percutaneous coronary intervention
POC: point of care
PROBE: prospective, randomized, open-label, blinded endpoint
RMSSD: root mean square of the differences in successive pairs of RR intervals
SDNN: standard deviation of normal RR intervals
SNS: sympathetic nervous system
T2DM: type 2 diabetes mellitus
