# Supplementary figures and images for: α-Glucosidase inhibitor miglitol attenuates glucose fluctuation, heart rate variability and sympathetic activity in patients with type 2 diabetes and acute coronary syndrome: a multicenter randomized controlled (MACS) study
Source: Cardiovasc Diabetol. 2017 Jul 6;16:86. doi: 10.1186/s12933-017-0571-1 (PMC5501494; doi:10.1186/s12933-017-0571-1)

## Slide 1
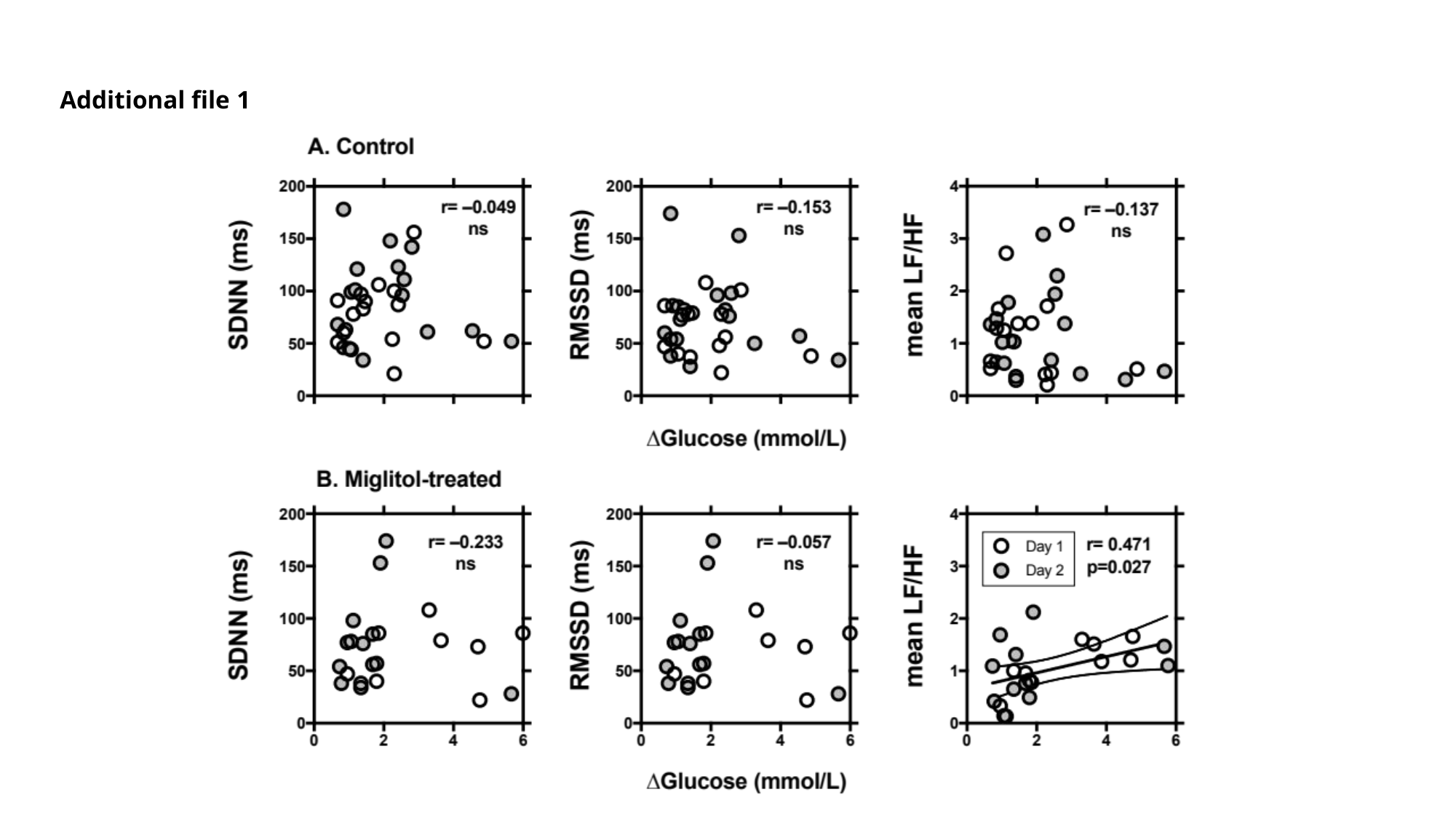

Additional file 1

Supplement: Supplementary file 1 — Additional file 1. Correlations of difference (∆Glucose) in 24-hour glucose levels with heart rate variability parameters. [file 12933_2017_571_MOESM1_ESM.pptx]

## Slide 1
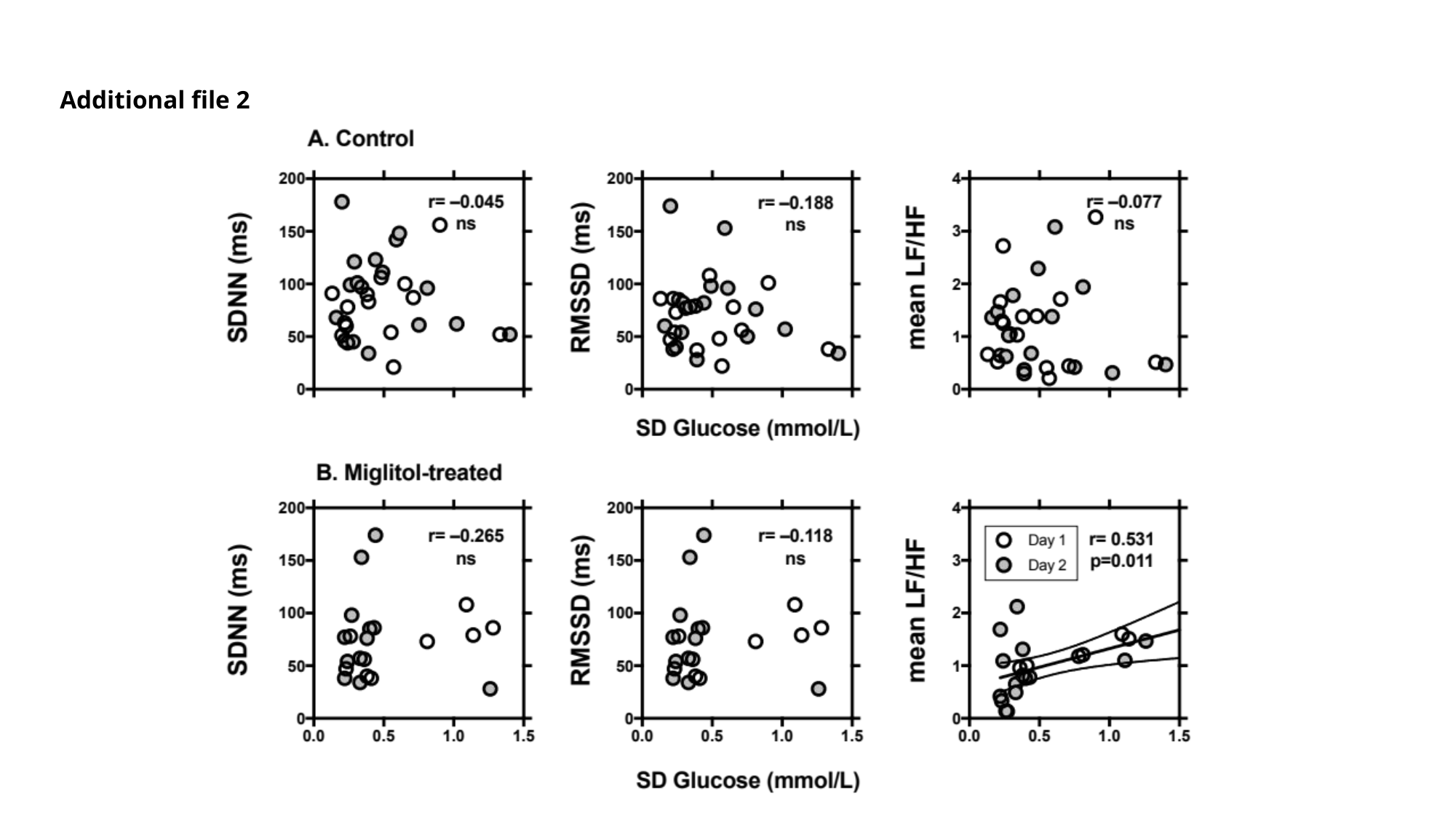

Additional file 2

Supplement: Supplementary file 2 — Additional file 2. Correlations of standard deviation (SD Glucose) in 24-hour glucose levels with heart rate variability parameters. [file 12933_2017_571_MOESM2_ESM.pptx]
